# Remembering Alan Magill

**DOI:** 10.4269/ajtmh.935mem

**Published:** 2015-11-04

**Figure:**
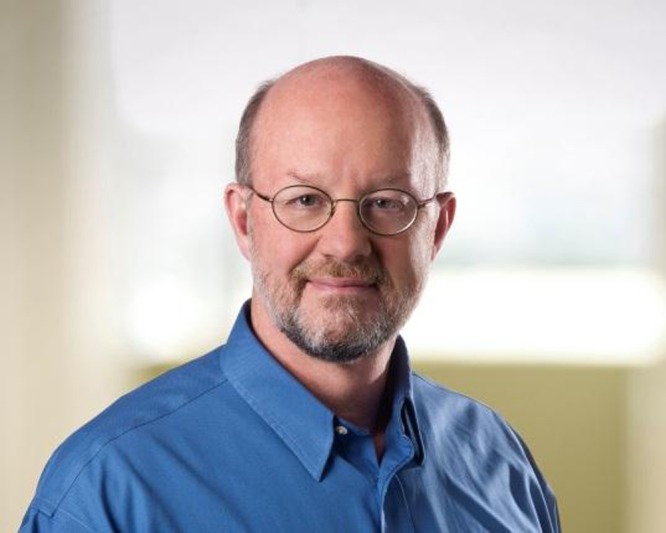
**Alan Magill, 1953–2015**

We note with profound sadness the unexpected death of Alan J. Magill, MD, FASTMH, immediate past-president of the American Society of Tropical Medicine and Hygiene (ASTMH), on September 19, 2015 outside Seattle.

Alan was president of our Society in 2014. He served with distinction, bringing to this elected position his characteristic enthusiasm, optimism, and focus. A noteworthy feature of his presidential year was the Ebola outbreak in Africa; he led efforts by the Society to address this horrific epidemic, including support for urgent scientific work and communication, and the need to respect the human rights aspects of the epidemic. He was greatly interested in the future of the Society, in particular its attention to its core missions of sharing scientific evidence, informing health policy, fostering career development, recognizing excellence, and advocating for investment in tropical medicine research. His efforts allowed us to move noticeably closer to the Society's vision of a world free of tropical infectious diseases.

Alan was the director of the Malaria Program of the Bill & Melinda Gates Foundation. Under his leadership the Foundation made great strides in its ambitious goal to eradicate malaria. Although there is a long way to go, we have clearly turned the tide on malaria and we have Alan to thank as one of the leading engineers of this success story. More broadly, he was an enthusiastic educator about infectious diseases, and a global leader in the fight against them.

Following his medical training, Alan served with distinction for 26 years in the U.S. Army Medical Corps, with a focus on clinical infectious diseases and research. His activities included clinical work in Germany, leading the Parasitology group at the U.S. Naval Medical Research Unit No. 6 Peru, directing research efforts to identify important impacts of malaria and leishmaniasis in U.S. military campaigns, and leadership in infectious diseases research at the Walter Reed Army Institute of Research. He was then a program leader at the Defense Advanced Research Projects Agency before moving to the Bill & Melinda Gates Foundation. Among Alan's many accomplishments were leading multiple research efforts at the U.S. Department of Defense, including the development of multiple diagnostics and treatments for malaria and leishmaniasis; discovery of a novel parasitic disease, viscerotropic leishmaniasis; serving as lead editor on the ninth edition of *Hunter's Tropical Medicine and Emerging Infectious Diseases*, a leading textbook; serving as coeditor for *CDC Health Information for International Travel* (the Yellow Book), the leading resource for travel medicine information in the United States; and authorship of numerous scientific papers and book chapters on malaria, leishmaniasis, travel medicine, and other topics in infectious diseases and military medicine.

Alan's passion, good humor, intellect, and drive will be greatly missed by the ASTMH. His impacts were huge, with broad contributions on tropical infectious diseases and travel medicine over many years; mentoring generations of clinicians, soldiers, and scientists; and leadership of enormous efforts to control and eradicate malaria and other infectious diseases. For our Society, Alan brought focus to our mission and vision. His contributions and sprit live on as we work to fulfill his goal of a world free of malaria and other serious tropical infections.

